# Recent Progressions in Applications of Bioactive Polysaccharides in Food and Health Sciences: A Comprehensive Review

**DOI:** 10.1002/fsn3.71482

**Published:** 2026-01-28

**Authors:** M V N L Chaitanya, Bahjat Alhasso, Wadhah Hasan Alkhazali, Ashok Kumar Bishoyi, Rami Oweis, S. Renuka Jyothi, Rishiv Kalia, Laxmidhar Maharana, Ashish Singh Chauhan, Hayder Naji Sameer, Ahmed Yaseen, Zainab H. Athab, Mohaned Adil, Asghar Narmani, Bagher Farhood

**Affiliations:** ^1^ School of Pharmaceutical Sciences Lovely Professional University Phagwara Punjab India; ^2^ College of Pharmacy Alnoor University Nineveh Iraq; ^3^ Ahl Al Bayt University Karbala Iraq; ^4^ Department of Microbiology, Faculty of Science, Marwadi University Research Center Marwadi University Rajkot Gujarat India; ^5^ Modern College of Business and Science Muscat Oman; ^6^ On Sabbatical Leave Jordan University of Science and Technology Irbid Jordan; ^7^ Department of Biotechnology and Genetics, School of Sciences JAIN (Deemed to be University) Bangalore Karnataka India; ^8^ Centre for Research Impact & Outcome, Chitkara University Institute of Engineering and Technology Chitkara University Rajpura Punjab India; ^9^ Department of Pharmaceutical Sciences Siksha 'O' Anusandhan (Deemed to be University) Bhubaneswar Odisha India; ^10^ Division of Research and Innovation, Uttaranchal Institute of Pharmaceutical Sciences Uttaranchal University Dehradun Uttarakhand India; ^11^ Collage of Pharmacy National University of Science and Technology Dhi Qar Iraq; ^12^ Gilgamesh Ahliya University Baghdad Iraq; ^13^ Department of Pharmacy Al‐Zahrawi University College Karbala Iraq; ^14^ Pharmacy College Al‐Farahidi University Baghdad Iraq; ^15^ Department of Medical Nanotechnology, School of Advanced Technologies in Medicine Tehran University of Medical Sciences Tehran Iran; ^16^ Department of Medical Physics and Radiology, Faculty of Paramedical Sciences Kashan University of Medical Sciences Kashan Iran

**Keywords:** bioactivity, biomedical applications, food and nutrition, polysaccharides

## Abstract

Nowadays, the incidence and mortality rates of diseases remain major challenges throughout the world. To stop these health‐threatening concerns, a safe diet plays a pivotal role. Polysaccharides, as natural biopolymers, possess remarkable potential in the development of safe and healthy foods to prevent the incidence of diseases and guarantee health. These abundant biopolymers have a high rate of practicality in the food industry and biomedical sciences. Polysaccharides can be found in various living organisms (including plants, seaweeds, animals, and microorganisms) and extracted by a number of advanced techniques. The anti‐inflammatory, immunomodulatory, hypoglycemic, hypocholesterolemic, anticoagulant, antiviral, antimicrobial, and antioxidant activities of polysaccharides have considerable features in the food and health sciences. The applications of polysaccharides in food sciences are mainly owing to ameliorating fatigue, preserving the microflora of the gastrointestinal tract, and providing health for the gut in living organisms. Moreover, polysaccharides have practical applications in pharmaceutical (drug delivery systems) and biomedical (regenerative medicine) sciences, which pave the way for the treatment of diseases. This comprehensive review highlights the potential applications of polysaccharides in food and health sciences.

## Graphical abstract

Polysaccharides play a pivotal role in the development of safe and healthy foods. They ameliorate fatigue, preserve the microflora of the gastrointestinal tract, and provide health for the gut in living organisms. These biopolymers are applied for the fabrication of breakthrough drug delivery systems and potential platforms in regenerative medicine.

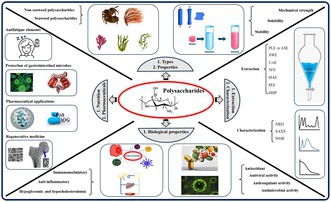

## 1 Introduction

Nowadays, human health is affected by various environmental threats (Biswas et al. 2024; Chen et al. 2025). The incidence of diseases has a high rate compared with the last decades, which is correlated with environmental factors, such as the type of food and the quality of nutrition. There is a direct correlation between human health and nutrition (Denning 2024; Feng et al. 2025; Amirishoar et al. 2023). For instance, the induction of cancer incidence is one of the main results of using unhealthy foods, high fatty acid‐containing nutrition, and harmful primary materials in cooking (Saadh, Allela, et al. 2025; Han et al. 2025; Oliero et al. 2024; Narmani et al. 2025). Therefore, increased production and use of bio‐based materials is considered the most promising strategy to produce healthy foods and guarantee human beings' health in recent years (Narmani et al. 2025; Yang, Chang, et al. 2024).

The primary source of healthy foods can directly be extracted from biomass, like proteins, lipids, and more importantly polysaccharides. Among these biopolymers, polysaccharides have attracted the attention of researchers and scientists for biomedical products and food science (Li, Ahmed, et al. 2025; Yazdi et al. 2022; Matei et al. 2025; Narmani, Kamali, Amini, Salimi, et al. 2018). Polysaccharides, as natural active ingredients, are made of more than 10 monosaccharides linked with each other by glycosidic bonds (Ju et al. 2023; Narmani, Ganji, et al. 2023). There are two major types of polysaccharides: homopolysaccharides (made of the same kind of residues) and heteropolysaccharides (made of multiple kinds of residues). These biopolymers possess ketone groups or aldehyde groups and are divided into several types based on their origin, like chitosan, dextran, pullulan, alginate, hyaluronic acid, etc. (Zhao et al. 2025; Wang, Wang, et al. 2024; Almajidi et al. 2024). These biopolymers have lots of physicochemical and mechanical characteristics, including stability, surface functional groups, surface modification, variable molecular weight, appropriate tensile strength, and so forth, which facilitate their practicality. Appropriate biocompatibility, biodegradability, accessibility, and affordability are other potential aspects of polysaccharides (Ramadan et al. 2025; Qiu et al. 2025; Gong et al. 2022). Polysaccharides are appropriate for biomedical and nutritional applications, such as immunomodulation, antioxidant, antimicrobial, anticancer, anti‐diabetes, liver protection, hypoglycemic, anti‐obesity, intestinal antiinflammation, and anti‐fatigue functions (Goushki et al. 2024; Xu et al. 2024; Almajidi et al. 2023). This review paper summarizes the source, extraction, characterization, physicochemical aspects, and biological properties of polysaccharides and emphasizes the pivotal role of polysaccharide‐based biomaterials in nutrition, fatigue, and gastrointestinal microbes. Besides, the importance of polysaccharides in pharmaceutical and biomedical sciences is surveyed in the overview.

## 2 Classification of Polysaccharides Based on Their Sources

### 2.1 Seaweed Polysaccharides

#### 2.1.1 Brown Seaweed Polysaccharides

##### 2.1.1.1 Alginates

Alginate is a kind of biocompatible anionic polysaccharide that is found in the intercellular matrix and cell walls of brown seaweed. This polysaccharide is made of a linear chain of monosaccharides consisting of α‐L‐guluronic acid (G block) and β‐D‐mannuronic acid (M block), which are linked with each other by (1 → 4)‐glycosidic bonds (Qamar et al. 2024). There are three patterns of structural block for alginate, including homo‐polymeric G blocks chain, homo‐polymeric M blocks chain, and hetero‐polymeric chains with randomized G and M monomers. Alginate with homo‐polymeric G block chain possesses higher hydrophilicity compared with M block alginate, which is owing to the capability of G block in the formation of hydrogen bonds (Pengyan et al. 2021). This alginate has a rigid structure as well. This polysaccharide is used for the formation of gel in tissue engineering, and the presence of G‐ or M‐blocks can affect the rigidity of the hydrogel. The rigidity based on G‐blocks is owing to forming the 2/1 buckled helical conformation and cooperative binding of Ca^2+^, while M blocks have a more flexible ribbon‐like conformation and less cooperative Ca^2+^ binding, which is suitable for high flexibility and less rigidity (Kopplin et al. 2021). The gel rigidity of alginic acid polymers is based on the following order: homo‐polymeric G blocks > homo‐polymeric M blocks > hetero‐polymeric MG blocks (Tammina et al. 2025). A low ratio of M/G results in high gel strength, whereas a high ratio of M/G leads to higher flexibility after fabrication of gels. Alginates with higher M/G ratios are applied for chronic wound healing by stimulating the secretion of cytokines from monocytes, while alginates with lower M/G ratios are used for the development of drug delivery systems (Ramos et al. 2018; Stavarache et al. 2024).

##### 2.1.1.2 Laminarins

Laminarin, a low molecular weight (Mw) polysaccharide (5 kDa), is mentioned as the main component of brown seaweed (up to 35% of content). This biopolymer is composed of (1 → 3)‐linked β‐D‐glucopyranose monomers with a variable number of β‐(1 → 6)‐intrachain linkages and 6‐*O*‐branching (Karuppusamy et al. 2024; Cheong et al. 2025). Various elements, like harvest time, species, and habitat of seaweeds, can affect the content percentage of laminarin. Regarding species type, while this polysaccharide is highly found in *Saccharina* spp. and *Laminaria* spp., some species (such as *Fucus* spp., *Undaria* spp., and *Ascophyllum* spp.) have low content of laminarin. Laminarin has two polymeric chains in its structure: G‐chain and M‐chain. The M‐chain possesses D‐mannitol at the reducing end of the chain, whereas the G‐chain does not. This polysaccharide has immunomodulatory and high blood compatibility, which makes it a potential candidate for pharmaceutical applications (Yu et al. 2025).

##### 2.1.1.3 Fucoidans

This anionic biopolymer is a type of seaweed polysaccharide composed of fucose units and sulfate ester groups. These polysaccharides contain additional monosaccharides and functional groups in their structures, including mannose, galactose, glucose, xylose, uronic acids, and acetyl functional groups (Zhang and Thomsen 2021). This polysaccharide is found in the primary cell wall of brown seaweed. The biological role of fucoidans is to preserve a moisture medium for seaweed in low‐tide circumstances and enhance their desiccation tolerance in harsh conditions. Various factors can modify the concentration of fucoidans in seaweed (ranging from 10% to 20% despite some exceptions, such as 46.6% fucoidan in *Laminaria digitata* ) (Zhang and Thomsen 2021; Shao and Duan 2022); for example, algal species, reproduction periods, and environmental elements (temperature, seawater salinity, oxygen level, etc.) may alter their biological concentrations. These polysaccharides have been developed for pharmaceutical science and drug delivery systems (Zhou et al. 2025).

#### 2.1.2 Red Seaweed Polysaccharides

##### 2.1.2.1 Agars

Agar, a linear seaweed polysaccharide, is composed of *α*‐(1 → 3)‐*D*‐galactopyranose and *β*‐(1 → 4)‐linked 3,6‐anhydro‐*L*‐galactopyranose monomers. This biopolymer has alternative sulphate groups at the C‐6 position (Nishinari and Fang 2017). Agar consists mainly of agarose and agaropectin. This polysaccharide has appropriate hydrocolloid characteristics and is mainly extracted from *Pterocladiella capillacea*, *Gracilaria* spp., and *Gelidium* spp. If the linear chain of agar possesses α‐(1 → 4)‐linked 3,6‐anhydro‐D‐galactopyranose residues, its hydrocolloid feature can be maximized (Nishinari and Fang 2017; Shah et al. 2024). The sulphation degree of agar can modify the anionic charges of agar and its solubility and stability features. A low degree of sulphation can result in neutral agarose, while agaropectin is the result of a higher sulphation degree. There is a direct correlation between the agarose gel with high viscosity and the presence of α‐(1 → 4)‐linked 3,6‐anhydro‐D‐galactopyranose monomer in the structure of the polymer. On the other hand, the presence of higher values of pyruvate and acetate substitutions in agaropectin is associated with low hydrophilicity. This polysaccharide is used in gel electrophoresis, immunology, microorganism culture, gene mapping, and immobilized technology (Shah et al. 2024; Sudhakar et al. 2025).

##### 2.1.2.2 Carrageenans

Carrageenan is made of disaccharide units of 3,6‐anhydro‐galactose and *D*‐galactose containing alternative 4‐*α*‐*D*‐galactose and 3‐*β*‐*D*‐galactose linkages in the backbone (Cadar et al. 2025). The commercial form of carrageenans is extracted from *Eucheuma denticulatum* and *Kappaphycus alvarezii* (Daet et al. 2025). This polysaccharide can be modified by substitution with methyl, ester sulfate, and pyruvate (Otero et al. 2023). Carrageenans, together with agar, are categorized as sulfated galactans. Carrageenans are classified into three types based on the structural composition and sulfate content, including the beta (β) type (beta (β), gamma (γ), omega (ω) and psi (ψ) carrageenans), the kappa (κ) type (kappa (κ), mu (μ), iota (ι) and nu (ν) carrageenans), and the lambda (λ) type (lambda (λ), alpha (α), delta (δ), theta (θ) and xi (ξ) carrageenans) (Alshammari et al. 2025). The main commercial types of carrageenans are extracted from *Kappaphycus alvarezii* (type of κ‐carrageenans) and *Eucheuma denticulatum* (type of ι‐carrageenans). These commercial types are developed for the fabrication of brittle gels (κ‐carrageenans) and soft gels (ι‐carrageenans). Various ratios of ι‐carrageenans and κ‐carrageenans in seaweed species lead to medium rheological features (Alshammari et al. 2025; Martín‐del‐Campo et al. 2021).

#### 2.1.3 Green Seaweed Polysaccharides

##### 2.1.3.1 Ulvans

This polysaccharide is a kind of hydrophilic dietary fiber extract from *Ulva* species. This sulfated polysaccharide is composed of sulfated rhamnose, uronic acid (iduronic or glucuronic acids), and xylose (Akter et al. 2024). The major sources of marine Ulvans include *Ulva rigida* , *Ulva lactuca* , and *Monostroma* spp. This polysaccharide possesses high Mw, ranging from 660,000 to 760,000 g/mol, and is composed of 77% to 79% g/g carbohydrates (Kraithong et al. 2024). Ulvans have indicated antioxidant, anticoagulant, anticancer, antihyperlipidemic, antiviral, antimicrobial, and immunomodulatory functions, which made them potential candidates for biomedical and pharmaceutical applications (Kraithong et al. 2024; Pari et al. 2025).

### 2.2 Other Polysaccharides With Different Origins

There are other types of practical saccharides that originated from different routes, including marine polysaccharides and plant polysaccharides. In what follows, these polysaccharides are going to be explored. Practical marine polysaccharides include chitosan, dextran, and hyaluronic acid, which have indicated potential practicality in biomedical science and the food industry. (i) *Chitosan* is composed of randomly distributed β‐(1‐4)‐linked D‐glucosamine and N‐acetyl‐D‐glucosamine units (Udupi et al. 2025). This biopolymer is obtained from the deacetylation of chitin. Chitosan is approved by the Food and Drug Administration (FDA) for a wide variety of biomedical applications, such as antioxidant function, anticancer activity, and additives in the cosmetic and food industries. The biological activity of chitosan can be manipulated by various physicochemical factors, such as molecular weight and deacetylation degree (Sivasuriyan et al. 2025; Jiang, Althomali, et al. 2023). The presence of amino and hydroxyl groups on the surface of chitosan gives an opportunity for this polysaccharide to be modified and improved in terms of biomedical applications. (ii) *Dextran* is another FDA‐approved polysaccharide made of α‐D‐glucose units with α‐(1‐6) glycosidic linkage between the units (Li et al. 2025). This biomaterial has practical applications in the fabrication of active drug delivery systems, the reduction of fast body clearance of bioactive compounds, the development of scaffolds in regenerative medicine, using as stabilizers in the food industry, and acting as emulsifiers in chemical science (Wang, Wu, et al. 2025). (iii) *Hyaluronic acid*, a kind of FDA‐approved linear polysaccharide for biomedical applications, is made of N‐acetyl‐D‐glucosamine and D‐glucuronic acid units (Ruan et al. 2025). This biopolymer is one of the crucial structural elements for skin and extracellular matrix and has potential applications in targeted drug delivery against cancer cells since it is applied as a targeting agent of CD44 receptors on the surface of cancer cells (Guo et al. 2025).

Plants have been mentioned as potential biological sources for the extraction of polysaccharides. These biomaterials have appropriate availability, biocompatibility, biodegradability, and numerous biological functions similar to seaweed polysaccharides, which introduce them as remarkable bioactive for biomedical science and the food industry. Some of the major plant polysaccharides include *G. lucidum* (Lingzhi) polysaccharides, which originated from a traditional Chinese plant, called *G. lucidum* . This polysaccharide has antioxidant, anticancer, and immunomodulatory functions (Xu et al. 2017). *Bletilla striata* polysaccharide extracted from *B. striata* has indicated practical applications in the cosmetic industry and wound healing, such as the treatment of chapped skin (Li, Han, et al. 2024). *Panax notoginseng* polysaccharide originated from *P. notoginseng* and has antioxidant, antiaging, and neuroprotective performances in the human body (Yu et al. 2024). Other practical polysaccharides include *Plantaginis semen* (can serve as an adjuvant, antioxidant, and immunomodulatory agent), *Lycium barbarum* (with potential antioxidant and anticancer activities), and *Radix hedysari* (may act as an antioxidant, anticancer, and antidiabetic bioactive) (Mo et al. 2022; Zhang, Wang, Xin, et al. 2024).

## 3 Physicochemical Properties of Polysaccharides

Polysaccharide is a kind of long‐chain carbohydrate made of monosaccharide units that are linked to each other through glycoside linkages. The type of monosaccharide, molecular weight, functional groups, surface charge, chain length, and branching can be used in the classification of polysaccharides (Dattilo et al. 2023). The glycoside linkages between the carbon atom of the donor and acceptor in monosaccharides are the distinguishing feature of polysaccharides compared with other biomolecules. The surface charge of polysaccharides is one of the major determinants in categorizing polysaccharides (Dattilo et al. 2023; Putro et al. 2023). Based on the surface charge, polysaccharides are divided into two types: positively charged polysaccharides and negatively charged polysaccharides. Chitosan is a type of positively charged polysaccharide (due to amino groups), whereas alginate, heparin, hyaluronic acid, and pectin are categorized as negatively charged polysaccharides (due to carboxylic acid groups) (Putro et al. 2023). Surface engineering (surface modification and surface functionalization) of polysaccharides can be implemented by the presence of surface functional groups; for instance, polysaccharides can be modified based on carboxymethylation, carbonylation, and sulfation processes which can give different functions for polysaccharides and change their physicochemical (stability, solubility, mechanical strength, etc.) and biological (cell targeting, non‐immunogenicity, etc.) performances (Putro et al. 2023; Chen, Liu, Shen, et al. 2023). For example, although polysaccharides have appropriate serum stability in the circulatory system, their stability could be further improved after surface modification with biocompatible molecules and moieties, such as polyethylene glycol (PEG) (Chen, Ge, et al. 2024). The glycosidic bond configuration and position are important for preventing serum hydrolysis; for example, β‐linkages (including β‐1,3, β‐1,4, and β‐1,6) are weakly hydrolyzed by human glycosidases, which are developed to cleave α‐linkages (especially in glycogen and starch) (Cotas et al. 2025). Besides, the surface charge of polysaccharides repels the human enzymes, which prevents the enzymatic affinity of the enzyme to polysaccharide and its degradation (Kudzin et al. 2025). Regarding the solubility, the presence of surface functional groups on the structure of polysaccharides could improve electrostatic repulsive forces and improve their solubility. Moreover, steric repulsion forces could further elevate the solubility of these biopolymers (Qiu et al. 2025). The mechanical strength of polysaccharides is correlated with their backbone and chain conformation, glycosidic linkage, hydrogen bonding, and crystallinity. The linear and unbranched, β‐1,4 linkage in equatorial position, high inter‐ and intramolecular hydrogen bonds, and high crystallinity (50% to 90%) result in high mechanical strength (Peesapati et al. 2024; Chen, Zhang, et al. 2024).

Some polysaccharides, like cellulose and starch, have monosaccharide‐storing roles in living organisms and act as sources of energy, while others (like chitosan, hyaluronic acid, and chondroitin sulfate) are involved in providing mechanical strength and serve as structural elements (Chen, Liu, Shen, et al. 2023). The physicochemical properties of polysaccharides play a pivotal role in their bioactivity, drug conjugation efficiency, drug encapsulation efficiency, mechanical strength, degradation rate, and pharmacokinetics and pharmacodynamics (Zhang, Palanisamy, et al. 2025b). Polysaccharide carriers can transport through the intestinal lymphatic pathway and penetrate into target tissues and cells, which is an important issue in their bioactivity and treatment function. The bioactive‐entrapped polysaccharides can block the first‐pass metabolism and ignore P‐glycoprotein‐mediated efflux pumps in target cells (Zhang, Palanisamy, et al. 2025b; Zhang et al. 2023). Polysaccharides decrease the fast clearance of bioactive compounds from the circulatory system. On the other hand, these biopolymers can form hydrogels, aerogels, globular carriers, scaffolds, and composites which, in turn, enhance their practicality in disease treatment, regenerative medicine, and the food industry. These forms can be administered by various routes, such as oral, subcutaneous (SC), intravenous (IV), transdermal, intraperitoneal, nasal, etc., which demonstrates the applicability of polysaccharides in biomedical science (Li, Ahmed, et al. 2025; Ramezani et al. 2024).

## 4 Extraction and Characterization of Polysaccharides

### 4.1 Extraction Procedures

The applied procedure plays a significant role in the quality of extraction of polysaccharides since a lot of physicochemical characteristics of polysaccharides, such as stability, solubility, mechanical strength, availability of functional groups, etc., are directly correlated with the quality of extraction (Banerjee et al. 2023; Wen et al. 2025). The mentioned physicochemical properties have indicated a momentous impact on the biomedical and nutritional function of polysaccharides. During the last decades, various conventional approaches have been developed for the extraction of polysaccharides, which are nominated based on the process of extraction (such as liquid–liquid and solid–liquid); however, these procedures have several drawbacks that decrease their efficiency (Wu et al. 2025). For example, conventional methods require prolonged extraction time, high‐tech equipment to prevent inefficiency of extraction, and hazardous acidic and alkali incubation, which restricts the use of these inefficient approaches. Moreover, unaffordability is mentioned as another challenge of these procedures (Ke et al. 2023). Therefore, the development of breakthrough modalities for the extraction of polysaccharides is undeniable to have enough efficiency in large‐scale processes. The principles of novel and efficient approaches, along with their merits, demerits, and biomedical applications, are listed in Table 1.

**TABLE 1:** Various types of methods in extractions of polysaccharides.

Procedure	Principles	Merits	Demerits	Applications	References
PLE or ASE	Efficient version of the liquid extraction method, having rapid diffusion of solvent, use of organic liquid solvents at high pressure (3.5–20 MPa) and temperature (50°C–200°C)	User‐friendly, cost‐effective, high efficiency, very fast and accurate	Not suitable for thermosensitive compounds, weak selectivity, need to high pressure	Polysaccharides can use for antioxidant functions and against various diseases	Sacramento et al. (2022)
SWE	Applying water under high pressure (10–60 bar) and temperature (100°C–374°C), decreased viscosity and increased diffusion can be applied for polar compounds	Efficient extraction rate, high yield, appropriate for polar molecules, reduced use of solvent	Less cost‐effectiveness	Polysaccharides can be applied for antioxidant activities	Ruthes et al. (2020)
UAE	Efficient approach, applying acoustic cavitation phenomenon during the extraction process	Appropriate for thermolabile compounds, user friendly, manipulatable, affordable, reproducible	Using prolonged sonication time and high ultrasound intensity, structural damage, unaffordability	Polysaccharides can be applied for antioxidant and anticancer functions	Hao et al. (2020)
SFE	Solute separation from a solid or liquid matrix by applying extracting solvents above or near their critical temperature and pressure (CO_2_), applying supercritical fluid at critical circumstances (31.1°C and 73.8 MPa) and high diffusion coefficient	An efficient procedure, more reliable, accurate, nontoxic, cost‐effectiveness, suitable chemical inertness	Prolonged extraction process, poor polarity of supercritical CO_2_, applying high pressure	Polysaccharides can be applied for antioxidant activities	Bai et al. (2024)
MAE	Applying high temperature due to the absorption of electromagnetic radiation (300 MHz to 300 GHz), enhancement of the pressure inside the cells, disruption of cell wall	Easy to sue, fast, accurate, cost‐effective, high rate of purity	Limitations regarding the size and humidity of sample, applying microwave power, using high pressure and solvent nature	Polysaccharides can be applied for antioxidant and anticancer functions	Wang, Li, Lu, and Liu (2024)
PEF	Use of electric pulses (ranging from 0.1–0.3 kV/cm to 20–80 kV/cm) to the biomaterials, creating pores and disruption of cell membrane and wall which results in release of intracellular content	Desirable for the extraction of heat‐sensitive compounds, lack of applying temperature, enhanced purity	Interferences caused by external electric field, pulse number, using high NaOH concentration	Polysaccharides can use for antioxidant functions and against various diseases	Li et al. (2020)
HHP	Applying high pressure and simulating the charged groups deprotonation, dissociation of non‐covalent bonds in the cell membrane and wall	Appropriate for thermosensitive components, accurate, having suitable solvent permeability, enhanced rate of mass transfer	Unaffordable, prolonged extraction process	Polysaccharides can be applied for antioxidant and anticancer functions	Ahmadi et al. (2022)

Abbreviations: ASE, accelerated solvent extraction; HHP, high hydrostatic pressure extraction; MAE, microwave‐assisted extraction; PEF, pulse electric field assisted extraction; PLE, pressurized liquid extraction; SFE, supercritical fluid extraction; SWE, subcritical water extraction; UAE, ultrasound‐assisted extraction.

### 4.2 Structural Characterization

There are several analytical approaches for the structural characterization of polysaccharides, among which X‐ray diffraction, small‐angle X‐ray scattering (SAXS), and nuclear magnetic resonance (NMR) are the most significant types. The principles of these analytical procedures are based on energy release for particular types of linkages, which can be used for ascertaining the angle of rotation in structural linkages of polysaccharides (Ahmadi et al. 2022; Uto 2025). Moreover, based on these analytical devices, the structure, conformation, and functional groups of polysaccharides are determined, which are the main parameters in classification, determining physicochemical properties (such as solubility, mechanical strength, stability, and crystallinity). For example, if a polysaccharide has various rotations at each monosaccharide linkage, it exhibits the random conformation of the polysaccharide, which could be due to interactions between and within chains.

#### 4.2.1 X‐Ray Diffraction (XRD)

This analytical approach is applied to ascertain the morphology phases (occurrence of amorphous/morphous structure), crystallinity degree, and crystalline microstructure of polysaccharides (Uto 2025). The principle of this procedure is based on the interference and diffraction of X‐ray beams after leaving the crystal which constitutes a biopolymer. XRD can give extra information regarding the filler and the monomeric orientation (such as biaxial orientation) of a biopolymer. The samples with well‐oriented structures and crystallinity can give high‐quality XRD graphs (Sepe et al. 2025). The starch films and powders, morphology phases of different gelforming polysaccharides (such as κ‐carrageenan, xanthan gum, etc.), crystallinity of the extracted polysaccharides from *Alpinia oxyphyllae fructus* in various solution conditions, and amorphous form of polysaccharide extracted from *Eucommia ulmoides* leaves are some examples of XRD in polysaccharide characterization (Wang, Ruan, et al. 2024; Liu et al. 2023). The main limitations of this analytical approach are insensitivity to characterize the semi‐crystalline or predominantly amorphous polysaccharides (such as starch, hemicelluloses, and pectins), forming various solvates/hydrates due to disordering the sheets after water/solvent effect, and poor scattering from light atoms (including C, H, and O) which results in weak data quality (Stanciu et al. 2025; Ogawa et al. 2022).

#### 4.2.2 Small‐Angle X‐Ray Scattering (SAXS)

The principle of this method is based on the irradiation of X‐ray beams and detecting the small angle in the structure of biopolymers. The principles of reciprocal law (Fourier space: correlating the distance *r* in real space with the scattering vector *q* in scattering space) are the major basis of the analytical technique (Garina et al. 2024). This analytical approach is a fast and accurate type of characterization. One of the prominent merits of the SAXS technique is the capability of surveying macromolecules in both solution and crystallized forms. The possibility of using soluble forms of naturally occurring biopolymers has valuable worth, especially for some biopolymers for which preparing their crystallized forms is difficult (Fu and Shi 2024). The structural and conformational properties of type II arabinogalactans extracted from jasmine tea, sweet potato starch gels reinforced with curdlan (a β‐(1,3)‐glucan), and decyl succinic anhydride‐modified pullulan micelles are some examples of characterized polysaccharides with the SAXS technique (Huang et al. 2025; Wang, Wei, et al. 2025). The major limitations of this method are weak resolution envelope models. Besides, this analytical technique has a deficiency in analyzing the atomic positions, detecting secondary structure motifs, measuring glycosidic linkage conformations, ambiguity in the interpretation of results of highly flexible or branched polysaccharides, and determining precise branching patterns (Sun et al. 2022). Difficulty in analyzing samples with heterogeneity/polydispersity and impurity is mentioned as another restriction correlated with this analytical approach (Fanova et al. 2024).

#### 4.2.3 Nuclear Magnetic Resonance (NMR)

This procedure is focused on main chains, side chains, and surface functional groups of polysaccharides and offers potential information about the molecular motion and the time‐dependent structure of polysaccharides (Falourd et al. 2024). NMR has indicated higher sensitivity for microscopic structures in a short range than other procedures (such as X‐ray scattering). On the other hand, the analytical information of NMR can be lost on the long range and higher orders, and establishing the spatial position of atomic groups is difficult. NMR requires a prolonged running time for sample analysis compared with SAXS (de Carvalho et al. 2025). Two examples of NMR‐based characterization of polysaccharides include bioactive polysaccharides extracted from the medicinal plant *Astragalus membranaceus* and scleroglucans (branched β‐glucans) generated by *Sclerotinia sclerotiorum* (Chen, Jiang, et al. 2023; Elsehemy et al. 2020). Some limitations of these analytical devices include signal overlapping and spectral complexity (due to repeating monosaccharide units), poor resolution for large polysaccharides (owing to short T₂ relaxation times), poor capability in detection of minor structural features, and solubility and sample preparation difficulty (since many polysaccharides have poor solubility) (Liu et al. 2025; Yang, Song, et al. 2025).

## 5 Biological Properties of Polysaccharides

### 5.1 Anti‐Inflammatory Activity

It has been indicated that polysaccharides have the potential to suppress pro‐inflammatory cytokines, cell growth, cell proliferation, cell migration, and criteria of tissue injury (Li et al. 2022). This function of polysaccharides plays a crucial role in anti‐inflammatory and anticancer pathways in living organisms. For instance, sulfated polysaccharides extracted from *Gelidium pacificum* (with a molecular weight of 28,807 Da and consisting of 7.1% xylose, 59.7% galactose, and 19.76% galacturonic acid) and polysaccharides of *Arctium lappa L*. (with a molecular weight of 4256 Da and composed of fructose, glucose, galactose, and arabinose in a ratio of 0.675:0.265:0.023:0.016) remarkably downregulated the expression levels of MyD88 (myeloid differentiation factor 88), TRAF6 (tumor necrosis factor receptor‐associated factor 6), and TLR4 (Toll‐like receptor 4) in target cells (*Arctium lappa L*. for colon Caco‐2 cells and *Gelidium pacificum* for THP‐1 cells) (Zeng et al. 2024). The overexpression of TRAF6 is correlated with elevated inflammatory responses (through stimulating the activation of T and B cells) and the induction of metastasis and tumor invasion in patients with cancer. On the other hand, MyD88 is engaged in stimulating the release of pro‐inflammatory factors from immune danger signals, while TLR4 induces the generation of inflammatory cytokines by the NF‐κB signaling pathway (Guangwei et al. 2022). Moreover, chitosan polysaccharide has demonstrated potential anti‐inflammatory responses, especially against colitis. Chitosan may suppress the expression of chitinase enzyme YKL‐40 in primary human macrophages, whereas it elevates the expression of tight junction proteins (occludin and claudin‐1) and regulates the function of colorectal barriers through signaling pathways (Alhamdi et al. 2025). Based on reports, high molecular weight chitosan (> 29.2 kDa) has anti‐inflammatory functions, and low molecular weight chitosan (≤ 29.2 kDa) is involved in proinflammatory performances (Chen, Liu, Dong, et al. 2023).

### 5.2 Immunomodulatory

Immunomodulation is one of the most prominent functions of polysaccharides. Polysaccharides have revealed considerable performance in stimulating the activation of macrophages, T and B cells, natural killer cells, and secretion of cytokines, which are correlated with altering the expression of IFN‐γ (interferon γ), TNF‐α (tumor necrosis factor α), and interleukins (especially IL‐2, IL‐4, IL‐6, and IL‐12) (Li, Ren, et al. 2024). These factors are engaged in the suppression of cancer. Polysaccharides reduce the expression and secretion of pro‐inflammatory and inflammatory cytokines. Polysaccharides decrease inflammation through maximizing the number of regulatory T cells, which can suppress inflammation (Han et al. 2024). The upregulation of biomolecules on the membrane of macrophages and dendritic cells results in stimulating the secretion of cytokines and IL‐2 and initiating the immunomodulation process against cancer. Chitosan‐based polysaccharides may induce the secretion of cytokines, like NO, TNF‐α, and IL‐6 from macrophages and stimulate their growth, proliferation, and phagocytosis (Han et al. 2024; Eswar et al. 2023). In addition, they induce macrophages by binding to mannose receptors, the cluster of differentiation 14 (CD_14_), TLR (TLR2 and TLR4), and the Dectin‐1 receptor on the surface of these immune cells (Eswar et al. 2023). Fucoidan‐based polysaccharides may activate the MAPKs (mitogen‐activated protein kinases) and NO (nitric oxide) and induce the RAW264.7 murine macrophage cells, resulting in stimulation of the innate immune response in malignant tumor cells (Chen et al. 2019).

### 5.3 Hypoglycemic and Hypocholesterolemic Functions

Polysaccharides have a lot of potential to be used for the treatment of various diseases, such as hypocholesterolemia, hyperglycemia, hyperinsulinemia, and insulin resistance, in clinical trials (Németh et al. 2025). The *Ganoderma atrum* polysaccharide is one of the most beneficial carbohydrates for the treatment of these diseases in diabetic rats using mg/kg concentrations. This polysaccharide does not indicate kidney damage in type II diabetics, which presents its safety and applicability (Wang, Wu, et al. 2024). Based on this work, low dose polysaccharide (25 mg/kg, bw), middle dose polysaccharide (50 mg/kg, bw), and high dose polysaccharide (100 mg/kg, bw) with high‐sugar and high‐fat (HSHF) diet fed for 12 weeks were applied for the treatment of type II diabetics. The administration of polysaccharide caused a significant reduction in blood glucose concentration, as the blood glucose was minimized by 7.79%, 11.71%, and 12.52% after treatment with low, middle, and high doses, respectively. Besides, polysaccharides can be employed as bioactive/drug carriers; for example, insulin‐entrapped dextran‐chitosan carriers prepared based on polyelectrolyte complexation approach are developed for oral administration in patients and indicated long‐term hypoglycemic effects (Sankaran et al. 2025). Other polysaccharides, such as chitosan, kefiran, and sulfated polysaccharides extracted from *Bullacta*, exhibited hypoglycemic and hypocholesterolemic activities. Suppressing the *α*‐glucosidase and *α*‐amylase enzymes related to insulin regulation may restrict polysaccharide digestion and reduce the absorption of glucose in diabetes (Moradi and Kalanpour 2019). Acarbose and plant extracts containing bioactive substances are mentioned as hypoglycemic drugs since they revealed α‐glucosidase inhibitory action, which is suitable for minimizing circulatory glucose (Yang, Yang, et al. 2025).

### 5.4 Anticoagulant Activity

Although all types of polysaccharides (from different sources, including plants, corals, fungi, algae, and shellfish (shrimp, crab, crayfish, etc.)) have demonstrated anticoagulant function, sulfated polysaccharides and sulfonation of the non‐sulfated polysaccharides (like sulfate‐modified chitosan) have revealed better anticoagulant function compared with non‐sulfated polysaccharides (Peipei et al. 2024). These polysaccharides are known as low molecular weight heparins. The high sulfate concentration of sulfated polysaccharides is directly correlated with their anticoagulant functions. Sulfated polysaccharides indirectly suppress factor Xa function by antithrombin III activity and directly suppress thrombin function, which is involved in coagulation (Holmes et al. 2023). Regarding non‐sulfated chitosan, there are two primary coagulation mechanisms that are correlated with charge neutralization and interparticle bridging. The surface positive charge of chitosan plays a pivotal role in both these processes (Zhang, Yan, et al. 2025).

### 5.5 Antiviral Activity

**FIGURE 1:**
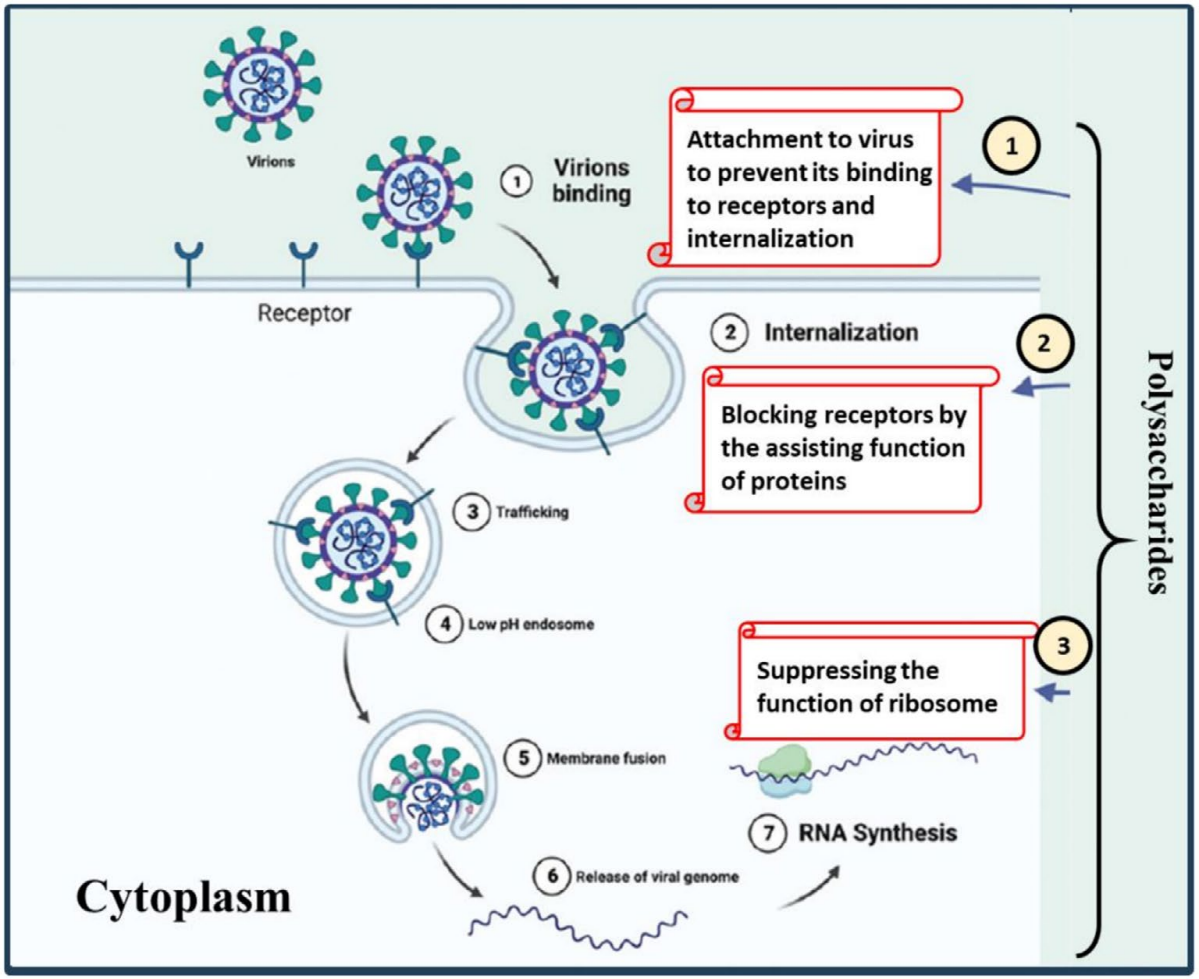
The possible antiviral mechanism of polysaccharides.

Polysaccharides have the potential ability to interfere with the duplication of enveloped viruses; for example, sulfated polysaccharides extracted from microalgae species can block the proliferation of human cytomegalovirus, human immunodeficiency virus (HIV), herpes simplex virus (HSV), respiratory syncytial virus, etc. (Deng et al. 2025). On the other hand, polysaccharides may strengthen the function of the immune system through the induction of the activity of macrophagocytes, which, in turn, maximizes the phagocytic performance of immune system cells and stimulates the secretion of IFN and antibodies (Hsu et al. 2024; Vasilakis et al. 2025). The possible antiviral function of polysaccharides is indicated in Figure 1.

### 5.6 Antimicrobial Activities

Polysaccharides have been depicted to have excellent antimicrobial function against bacterial pathogens, fungal pathogens, and viruses. Based on reports, fucoidan polysaccharide extracted from *Fucus vesiculosus* can suppress the growth of *Staphylococcus aureus* and *Listeria monocytogenes* strains (Gram‐positive bacteria) and inhibit the formation of their biofilms at 500 and 250 μg/mL concentrations, respectively (Arafa et al. 2022). The antibacterial function of alginates against *Agrobacterium tumefaciens* strain (Gram‐negative bacteria) is observed at a 3.90 μg/mL concentration, while it is attained at higher concentrations (100% at a 1.95 μg/mL) for Gram‐positive *Bacillus cereus* strain. The antifungal function of ulvan polysaccharide against *Aspergillus flavus* (91% at 10 mM concentration) and *Rhizopus stolonifera* (89% at 5 mM concentration) strains is indicated for samples extracted from *Ulva lactuca* (El Fayoumy et al. 2022). The fungicide‐loaded chitosan‐carrageenan carrier demonstrated antifungal activity (by 100%) against *Septoria lycopersici* and *Sclerotinia sclerotiorum* at 1 mg/mL concentration. The MIC (minimum inhibitory concentration) of chitosan oligosaccharides on the *Propionibacterium acnes* bacterial strain is not only observed at 32 to 64 μg/mL, but also in the inhibition of biofilm formation of *Staphylococcus aureus* (Kim et al. 2017). The surface function group of polysaccharides is one of the main elements in their antimicrobial performances. For instance, the formation of electrostatic interaction between positive amino groups of chitosan and negatively charged teichoic acids (Gram‐positive bacteria) and lipopolysaccharides (Gram‐negative bacteria) can result in disruption of the cell wall and leakage of cytoplasmic content (Xia et al. 2025).

### 5.7 Antioxidant

Polysaccharides have potential antioxidant functions in living organisms. These biopolymers are involved in radical substitution reactions to block the generation of reactive oxygen species (ROS). The donation of hydrogen or electrons to oxygen molecules is the basis of the generation of free radicals inside cells, which may be blocked by the antioxidant function of polysaccharides (Dong et al. 2025). The antioxidant performance of polysaccharides can be carried out through three processes of radical substitution reactions: inhibiting initiation process (which is evaluated by assessment of power activity, total antioxidant capacity, and DPPH/ABTS free radical scavenging performance), suppressing branching and propagation process (which is assayed by metal element chelation capability), and blocking termination process (which can be evaluated by superoxide/hydroxyl radical scavenging ability) (Rodrigues‐Souza et al. 2022). The polysaccharides of *Padina pavonica* and *Codium isthmocladum* (containing fucan and galactan) have potential to blockage of ROS. *Sargassum carpophyllum* indicated concentration‐dependent DPPH/ABTS radical scavenging functions. The metal chelating activity was observed for polysaccharides extracted from *Gracilaria caudata* at a 4 mg/mL concentration (Makhlof et al. 2024; Alencar et al. 2019). Besides, a 1 mg/mL concentration of fucoidan polysaccharide of *Sargassum fusiforme* resulted in the termination process of the radical substitution reaction (Wang et al. 2020).

## 6 Application of Polysaccharides as Regulatory Nutritional Elements in the Human Body Function

Among polymers, polysaccharides (such as starches and celluloses), proteins, and a few types of nucleic acids (DNA and RNA) have formed numerous naturally occurring biomaterials in a wide variety of foods. Polysaccharides are mentioned as the most practical biopolymers in food science. Polysaccharides, essential dietary components, are divided into several types based on their origins, such as chitosan, starch, hyaluronic acid, cellulose, galactomannans, agars, pectins, carrageenans, alginates, gums, etc. (Lin et al. 2024). These biomaterials are abundant in nature and may be remarkably applied in food science, from preparation and packaging to necessary bulk food and nutrition. Moreover, polysaccharides are used as potential food additives due to their exceptional characteristics, like stabilizing, thickening, gelling, and emulsifying ability (Ying et al. 2025). The charge, surface functional groups, hydrophobicity, structural stability, aromatic structural configurations, and mechanical strength of polysaccharides are considered potential properties of these biopolymers. The polymerization degree of polysaccharides ranges between 103 and 104. These biomaterials are involved in energy storage, building precursor, structural function, and metabolism processes, which guarantee the health of living organisms (Lee et al. 2025). The commercially available hydrocolloids of polysaccharides are indicated in Table 2. Starch is one of the essential dietary components of macronutrients, which directly correlates with the healthy lifestyle of human beings. The major applications of polysaccharides in food sciences are classified into two basic categories: (i) stabilizing food microstructures through gelling, thickening, emulsifying, and foaming, as well as using processing aids (including cryoprotectants to increase freeze–thaw stability, drying aids, and encapsulant material). (ii) Physiological and biological functions correlated with health claims, such as inducing satiety, controlling cholesterol levels in the circulatory system, improving bioavailability, and providing antimicrobial activity (Sayas‐Barberá et al. 2022; Tong et al. 2025). Several practical examples of polysaccharides in food sciences are indicated in Table 3.

**TABLE 2:** Source and commercially available polysaccharides in food science.

Source	Subtypes	Polysaccharide
Botanical	Seeds, plants, trees, tree gum exudates, tubers	Starch, pectin, cellulose, konjac mannan (glucomannan), gum, karaya
Algal	Red and brown seaweeds	Agar, carrageenan, alginate
Microbial	Various fungal and bacterial strains	Xanthan gum, dextran, gellan gum, cellulose
Animal	Various types of mammalians	Gelatin, caseinate, chitosan, hyaluronic acid

**TABLE 3:** Polysaccharides as potential diet and antifatigue elements in food science.

Polysaccharide source	Composition	Comments and biomedical effects	References
Herba epimedii	Mannose, rhamnose, glucose, galactose, arabinose, and galacturonic acid	Extracted with distilled water at 80°C, oral administration in rat models at 50, 100, 200 mg/kg for 4 weeks, increase the body weight and induce the secretion of noradrenaline and creatine	Chi et al. (2017)
*Lepidium meyenii* (Walp)	Galactose, glucose, arabinose, mannose, rhamnose	Extracted with hot water at 90°C, intragastrical administration in mice at 25, 50, 100 mg/kg bw/day dosage for 30 days, increase the body weight in mice and average swimming speeds, enhance GSH‐Px and CK, reduce the LDH and BUN	Tang et al. (2017)
Pumpkin	Glucose backbone with branching points of (1–3,4)‐linked‐ glucose and terminal glucose as side chain	Hypoglycemic activity in streptozotocin induced rat islet *β*‐cells at 250, 500 and 1000 μg/mL concentrations for 24 h of incubation in in vitro conditions	Chen Shan (2019)
*Pleurotus tuber*	Mushroom sclerotial polysaccharides	Immunity regulatory function, intraperitoneal administration into the mice to induce sterile peritonitis	Wong et al. (2011)
Chinese yam (*Dioscorea opposita Thunb*.)	Rhamnose, glucuronic acid, glucose, galactose, and arabinose	Water‐extracting and alcohol precipitating technique, orally administrated in Swiss mice at 100 mg/kg dosage for 14 days, increase body weight and SOD, decrease IL‐lβ, MDA, BUN, LDH	Wang et al. (2021)
Ginseng	Rhamnose, galactose, glucose, arabinose	Extracted under reflux extraction with 95% ethanol and hot water, orally administrated in male Kunming mice at 25, 50, 75 mg/kg dosage for 30 days, induce the proliferation of T or B lymphocytes, decrease BUN, LA, and MDA	Jiao et al. (2021)
*Lycium barbarum*	Arabinose, ylose, glucose, galactose	Extracted in 95% ethanol and ultra‐pure water at 90°C, intragastrical administration at 0.5, 2, 4 Se/kg/day dosage for 30 days, increase liver and muscle glycogen and SOD, decrease BUN, BLA, and MDA	Liu et al. (2021)
*Inonotus obliquus*	Mannose, glucose, galactose, xylose, and arabinose	Extracted in hot water, orally administrated in male Kunming mice at 50 mg/kg/day dosage for 30 days, decrease BLA, BUN, LDH, and 5‐HT	Zhang et al. (2020)

### 6.1 Polysaccharides as Antifatigue Elements

#### 6.1.1 Affecting Energy Metabolism

Energy metabolism is a crucial element for health. There are two main sources of energy substances for the normal function of the muscular system, including glycogen and fat, which can be used for the production of an applicable energy supply (ATP) in various intracellular activities (Zheng et al. 2025). Daily activities may slightly decrease the values of ATP, glycogen, and fat. Extreme daily activity and exercise‐induced fatigue, which is the result of continuous ATP consumption and lack of sufficient energy supply, can be reduced by the activities of polysaccharides since they are the primary source of energy during extreme daily activity and exercise. Polysaccharides serve as regulatory systems for energy metabolism through enhancing ATP content, adjusting lipid metabolism, regulating glycogen metabolism, and stimulating mitochondrial activities (Zheng et al. 2025; Wang, Wang, et al. 2025). The regulating and adjusting energy state could be sensed by adenosine 5′‐monophosphate‐activated protein kinase (AMPK). AMPK regulates glucose homeostasis as it initiates the commencement of catabolic pathways to generate ATP. The phosphorylation of AMPK may induce the activation of peroxisome proliferator‐activated receptor‐gamma coactivator‐1alpha (PGC‐1α), induce the resistance of muscle fibers to fatigue, and stimulate the production of ATP for the contraction of muscles. The *Panax ginseng C. A. Meyer* polysaccharide is applied as a supplement to boost the uptake of glucose and regulate glycolysis in animal models by overexpression of AMPK, phosphorylated‐AMPK, PGC‐1α, and glucose transporter 4 (GLUT‐4), which are engaged in the function of muscle tissues (Yu et al. 2023; Spaulding and Yan 2022).

Polysaccharides may regulate the metabolism of fat and energy supply, especially during extreme activity. It is exhibited that polysaccharides reduce the consumption of glycogen via stimulating fat/lipid metabolism. The *Ganoderma lucidum* polysaccharide (molecular weight > 10 kDa) increases the endurance of athletes through elevating fat conversion during exercise since AMPK induces fatty acid oxidation and stimulates the absorption of glycogen (Ren et al. 2025). AMPK and lipid homeostasis‐related PPAR (peroxisome proliferator‐activated receptor) signaling play a pivotal role in the regulation of glycogen phagocytosis in skeletal muscles. Boosting mitochondrial function may reduce oxidative damage to the mitochondrial membrane and consequently decrease fatigue during extreme activities (He et al. 2022). Polysaccharides improve mitochondrial biogenesis and act against mitochondrial dysfunction through overexpression of NAD^+^/NADH and mitochondrial biogenesis which, in turn, minimizes oxidative stress in muscles (He et al. 2022; Ji et al. 2025).

#### 6.1.2 Minimizing Metabolite Accumulation

The omission of the accumulated metabolites, like lactate dehydrogenase (LDH), blood lactic acid (BLA), and blood urea nitrogen (BUN), which are generated during extreme daily activity or exercise can be facilitated by polysaccharides. These accumulated metabolites disrupt homeostasis and result in fatigue; for instance, anaerobic glycolysis during extreme daily activity/exercise induces the accumulation of LA, which leads to acidic conditions and a sense of fatigue (Zhang, Li, et al. 2025a). The function of LDH assists in generating sufficient ATP and aids the removal of LA, which reduces the rate of metabolic acidosis. It is shown that *Maca polysaccharides* and *Inonotus obliquus polysaccharides* may potentially elongate the exhausting swimming time in animal models and minimize the values of LDH, BLA, BUN, and liver glycogen, which results in anti‐fatigue function (Liu et al. 2024). On the other hand, ammonia concentration in skeletal muscles can be enhanced during a high rate of metabolism in extreme daily activities/exercise, which activates phosphofructokinase, suppresses the oxidation of pyruvate (to generate acetyl CoA), stimulates the generation of metabolites (such as LA and BUN), decreases body endurance, and causes fatigue (Liu, Li, et al. 2022). The Maca polysaccharides potentially reduce the accumulation of the produced metabolites (LDH, BUN, and LA). The hydrophilic polysaccharide extracted from *Semen cassia*, *Sarcodon imbricatus*, and *Codonopsis pilosula* has demonstrated remarkable anti‐fatigue function in animal models via minimizing the levels of metabolites (such as BUN and triglyceride) in the serum of the circulatory system (Kang et al. 2021).

#### 6.1.3 Interfering With Autonomic Neuromodulation

There is a direct correlation between fatigue and the function of neurotransmitters (hydroxtryptamine (5‐HT), noradrenaline (NA), dopamine (DA), gamma‐aminobutyric acid (GABA), and acetylcholine). Extreme physical activity/exercise results in fatigue which, in turn, affects the balance of the oxidation/antioxidant process in the human body and disrupts the function of the central nervous system (CNS) (Yang, Zhu, et al. 2024). There is a direct correlation between the concentration of 5‐HT neurotransmitters and fatigue in muscles. The polysaccharides extracted from *Inonotus obliquus* may reduce the concentration of 5‐HT and result in antifatigue function (Lei et al. 2024). *Spirulina platensis* polysaccharide could not only minimize the concentrations of 5‐HT and tryptophan hydroxylase‐2 (TPH2) but also induce the overexpression of serotonergic type 1B (5‐HT1B) in living organisms with high physical activity, which are involved in antifatigue activity (Zhu et al. 2020). Furthermore, the interaction of 5‐HT and DA plays a regulatory role in the development of exercise fatigue. Polysaccharides obtained from *Antrodia camphorata* may suppress the expression of 6‐Hydroxydopamine (6‐OHDA)‐induced ROSNLRP3 and support the activities of the dopaminergic neurons which are suitable for antifatigue performance (Han et al. 2020).

#### 6.1.4 Regulating Endocrine System

Some studies have indicated that fatigue can affect the function of secretory glands/endocrine cells and hormone secretion. Regulating hypothalamic feedback plays a prominent role in living organisms with extreme physical activities and long‐term physical fatigue (Zhou et al. 2023; Song, Zhang, et al. 2025). The hypothalamus‐pituitary–adrenal axis is involved in physical fatigue by adjusting the production and secretion of cholesterol and testosterone, which subsequently regulates the immunosuppression and chronic pain of patients with fatigue (Wang et al. 2022). *Phragmites rhizome* polysaccharide revealed the anti‐fatigue activity via inhibiting the over‐activation of the hypothalamus‐pituitary–adrenal axis and reducing the concentration of cortisol (Chung et al. 2019).

#### 6.1.5 Boosting Immune System Function

It is indicated that polysaccharides have potential anti‐inflammatory and immunomodulating activities. These biomaterials may engage in boosting the immune system response in patients with various diseases (Muroya et al. 2025). Since fatigue can damage the immune system, polysaccharides can serve as immunomodulatory agents to repair it. For example, polysaccharides extracted from *Dendrobium officinale* have indicated a regulatory impact on immune system performance by enhancing the cell variability of T and B immune cells and alleviating fatigue syndrome (Wei et al. 2017). This polysaccharide can enhance the serum levels of macrophages and T and B immune cells in the spleen of animal models as well. The polysaccharides of steamed ginseng may increase the number of T and B immune cells after extreme physical activities/exercise and repair the created injuries in the human body (Jiao et al. 2021). Chitosan‐based materials have demonstrated positive effects on the repairing and development of the spleen and lungs, the ratio of T cell/CD8^+^ T cell, the number of T and B immune cells, and serum level of cytokines (like TNF, IL‐2, IL‐10, etc.) which, in turn, leads to antifatigue function in individuals (Xiong et al. 2020). Table 3 presents the practical examples of polysaccharides as diet and antifatigue elements.

### 6.2 Polysaccharides and Gastrointestinal Microbes

There is a direct correlation between the health of the intestinal gut and the function of microbes and levels of polysaccharides in the gut. The primary types of microbiomes in the intestinal gut of the human body majorly include *Firmicutes* and *Bacteroidetes* strains, while some other strains, including *Actinobacteria*, *Verrucomicrobia*, and *Proteobacteria* strains, are found at low levels. These bacteria are responsible for 90% of the intestinal microbiome (Neri‐Numa et al. 2020; Guo et al. 2020). These strains of bacteria must be at an optimum level to avoid causing diseases such as colorectal cancer; for instance, bacteria may attack epithelial cells of the intestinal gut at high levels and cause chronic intestinal inflammatory responses (Song, Liu, et al. 2025). Higher levels of *Escherichia coli* (inducing expression of polyketide synthase and colibactin and causing genome damage) and *Enterotoxigenic B. fragilis* (inducing the expression of spermine oxidase (SMO), signal transducer and activator of transcription 3 (STAT3), and IL‐17‐dependent carcinogenesis) are linked with colorectal cancer and transformation of colon adenoma to adenocarcinoma, respectively (Mafe and Büsselberg 2025; Wei et al. 2024). *Fusobacterium nucleatum* may induce the release of inflammatory factors (including IL‐6, CXCL1, IL‐8, IL‐10, and IL‐18) and result in the suppression of various immune cells (such as natural killer (NK) cells, CD_4_^+^T/CD_8_^+^T helper cells, and dendritic cells [DCs]), which, in turn, cause colorectal adenocarcinoma. Moreover, *Fusobacterium nucleatum* is correlated with the inhibition of immune system cells by manipulating the function of myeloid‐derived suppressor cells (MDSCs), inducing differentiation of macrophages into the tumor‐promoting M2‐phenotype, and stimulating tumor‐associated neutrophils (TANs) (Jiang, Xie, et al. 2023; Cheraghpour et al. 2024).

On the other hand, polysaccharides can directly affect the composition or metabolism of the gut microbiome, through which the health of the gut can be guaranteed by eliminating pernicious bacterial strains and facilitating the beneficial bacterial strains. The carbon source for the growth and proliferation of bacteria can be prepared by polysaccharides (Chang et al. 2025; Khalaf et al. 2023). Polysaccharides isolated and purified from *Lycium barbarum* may considerably induce the growth and proliferation of beneficial bacteria (including *Lactobacillus acidophilus* and *Bifidobacterium longum* ) and probiotic genera (like *Akkermansia*, *Lactobacillus*, and *Prevotellaceae*) (Wang, Li, Zhang, et al. 2024). Moreover, polysaccharides of *Crataegus pinnatifida* may act as probiotics and stimulate the growth of *Bacteroides* strains ( *Bacteroides ovatus* , *Bacteroides thetaiotamicron*, and *Bifidobacterium longum* ) (Feng et al. 2024).

Regarding the decomposition and digestion of polysaccharides, saliva and gastric and small intestinal conditions cannot degrade and digest these biopolymers. They need enzymatic digestion in the human body. There are only 17 enzymes for the decomposition and digestion of polysaccharides, which are encoded by the human body genome (Zhang, Wang, Zou, et al. 2024). In order to have complete digestion of polysaccharides, the enzymes encoded by the microbes and their genomes are required. The degradation and modification of polysaccharides are the major roles of bacterial digestive enzymes, called carbohydrate‐activated enzymes (CAZymes). These enzymes are categorized into six types: polysaccharide lyases, glycosyltransferases, glycoside hydrolases, carbohydrate esterases, carbohydrate‐binding modules, and auxiliary activities (Guo et al. 2023). The polysaccharide lyases and glycosyltransferases are involved in the degradation of glycosidic bonds; glycosyltransferases break the glycosidic bonds between carbohydrates‐carbohydrates and carbohydrates‐non‐carbohydrates in the main backbone of the biopolymeric chain by consumption of H_2_O molecule, while polysaccharide lyases are involved in the degradation of long‐chained carbohydrates containing uronic acids by the β exclusion mechanism (Song et al. 2024). Glycoside hydrolases degrade the long‐chained polysaccharides into oligosaccharides to facilitate biodegradation. Carbohydrate esterases play a pivotal role in removing polysaccharide ester groups from the backbone of polysaccharides and attaching these groups to the side chains, which results in the degradation of polysaccharides to small molecules. Carbohydrate‐binding modules could bind to the backbone of branched polysaccharides and enhance the concentration of other digesting enzymes. The auxiliary activities are engaged in the deconstruction of lignin and oxidative decomposition of cellulose and chitin (Liu, Wang, et al. 2022). There are three major mechanisms for polysaccharide degradation based on the function of gut microbiota: starch utilization system (Sus), ABC transport system, and the multienzyme complexes system. The *Bacteroides* apply the Sus mechanism for the degradation of polysaccharides. This system is made of various subunits, including Sus E and Sus F (for recognizing the polysaccharides and accumulating them at the surface of the cell), Sus R (as a transmembrane regulatory protein which could recognize decomposition of polysaccharides), Sus D (binds to glycoside hydrolases which are involved in the degradation of polysaccharides into oligosaccharides), Sus C (as transmembrane transporter of glycoside hydrolases), and Sus A/B (involved in degradation of oligosaccharides into smaller oligosaccharides by glycoside hydrolases and polysaccharide lyases) (McKee et al. 2021). The ABC transporters and multienzyme complexes system are found in *Firmicutes* and *Ruminococcus champanellensis* , respectively, which facilitate the transportation and degradation of polysaccharides in the human gut (Masselot Joubert and Di Renzo 2025; Huang et al. 2024).

Polysaccharides play a pivotal role in the production of functional metabolites, such as organic acids as a result of microbiological fermentation. The production of short‐chain fatty acids (SCFAs; like acetate, propionate, and butyrate) is one of the major organic acids that provide energy for the growth and proliferation of intestinal epithelial cells, regulation of the immune system cells, and action against diseases (Xue et al. 2024). This capability of polysaccharides guarantees the function of intestinal barriers by inducing the expression of tight junction proteins (claudin‐1 and ZO‐1) and the mucus secretion of goblet cells to the intestine, which may apply to repairing injuries in the gut (Yan et al. 2024). Additionally, polysaccharides can have a momentous impact on the function, diversity, and number of microbiotas in living organisms. There are two types of correlation between polysaccharides and microbiota: (i) Growth‐inducing effects on microbiota by acting as nutrition. Polysaccharides can be hydrolyzed to oligosaccharides and monosaccharides and transform into SCFAs which, in turn, provide enough nutrition for growth, division, and vital intracellular functions (Fang et al. 2021; Tang et al. 2022). (ii) Adjuvant function of polysaccharides to control and regulate the number of microbiota in the gut. This relationship between the bacterial strains and polysaccharides (as nutritional and adjuvant elements) paves the way for the normal function of intestinal and colorectal pathways, which demonstrates the significant importance of polysaccharides in food and health sciences (Tang et al. 2022).

Another function of polysaccharides is the modulation of inflammatory response in the cancer microenvironment. It is revealed that polysaccharides can suppress cancer cell growth, duplication, and metastasis as well as induce the commencement of apoptosis and autophagy pathways in cancer cells, like colorectal adenocarcinoma (Chen et al. 2022). Microbiota dysbiosis is one of the major issues in inducing their pathogenicity, which can be arrested by the polysaccharides. Polysaccharides may decrease the values of lipopolysaccharides in serum, alter tyrosine and tryptophan biosynthesis, and modify the metabolism of bile acid, which plays a crucial role in the suppression of colorectal adenocarcinoma (Zhao, Liu, et al. 2024). By inducing the secretion of anti‐inflammatory cytokine IL‐10 and inhibiting the secretion of pro‐inflammatory cytokines (such as IL‐6, TNF‐α, IFN‐γ, etc.), polysaccharides could arrest IL‐6/STAT3 signaling pathway and prevent inflammatory responses in colorectal adenocarcinoma (Li, Zhang, et al. 2024). Polysaccharides are involved in the reduction of phosphorylation of MyD88, TLR4, and NF‐κB p65, and the regulation of iNOS, IL‐1β, and COX‐2, which subsequently block the macrophage infiltration and arrest inflammatory responses in the cancer microenvironment (Zong et al. 2022; Fan et al. 2024).

## 7 Application of Polysaccharides in Pharmaceutical and Biomedical Sciences

### 7.1 Pharmaceutical Applications

Polysaccharides have been applied for the fabrication of drug delivery systems (DDSs) to treat diseases, such as cancer. DDSs based on polysaccharides are considered a type of breakthrough technology in pharmaceutical science that can be developed in forms of pharmaceutical formulations against infectious diseases (including bacterial‐, viral‐, and fungal‐born infections) and non‐infectious diseases, including gastrointestinal and respiratory diseases, diabetes, cardiovascular disorders, cerebrospinal abnormalities, and cancer (Paiva et al. 2024; Khakinahad et al. 2022; Yousefi et al. 2020; Abadi et al. 2025; Hosseini et al. 2025). These novel formulations possess remarkable advantages over conventional approaches in the treatment of diseases; to put it in a more vivid picture, medicines/formulations applied for disease therapy in conventional modalities have various limitations, such as weak serum stability and solubility, poor absorption from the circulatory system and gastrointestinal gut, inefficient biodistribution, prolonged treatment time, nano‐targeted internalization (in normal cells), fast clearance by the kidney and liver, immunogenicity, unaffordability, and so forth, which reduce the treatment efficiency (Chatterjee et al. 2023; Shao et al. 2025; Fotouhi et al. 2021). As a result, the development of polysaccharide‐based DDSs paves the way for the efficient treatment of diseases. Among polysaccharides, chitosan, alginate, dextran, hyaluronic acid, β‐cyclodextrin, cellulose, and starch have been applied for the fabrication of novel DDSs. High drug loading capacity and sustained/controlled drug release profile are the most important properties of DDSs based on polysaccharides (Narmani et al. 2017; Solomevich et al. 2023; Narmani 2025; Saadh, Ahmed, et al. 2025). Controlled drug release is defined as systemized drug release in the target tissues/cells for a prolonged time to preserve the drug concentration at the determined period of time. Sustained drug release refers to long‐term drug release with a constant rate at the site of action, which guarantees the presence of the drug in the disease site. There are lots of elements, including the type of polysaccharide, viscosity of the polysaccharide matrix, strength of intermolecular forces between the drug and polysaccharide matrix, values of entrapped drug, the preparation process of drug entrapment, and hydrophilicity/hydrophobicity of the drug, which effectively affect the controlled/sustained drug release from polysaccharide‐based DDSs (Solomevich et al. 2023; Wang, Gu, et al. 2023; Narmani et al. 2025).

**FIGURE 2:**
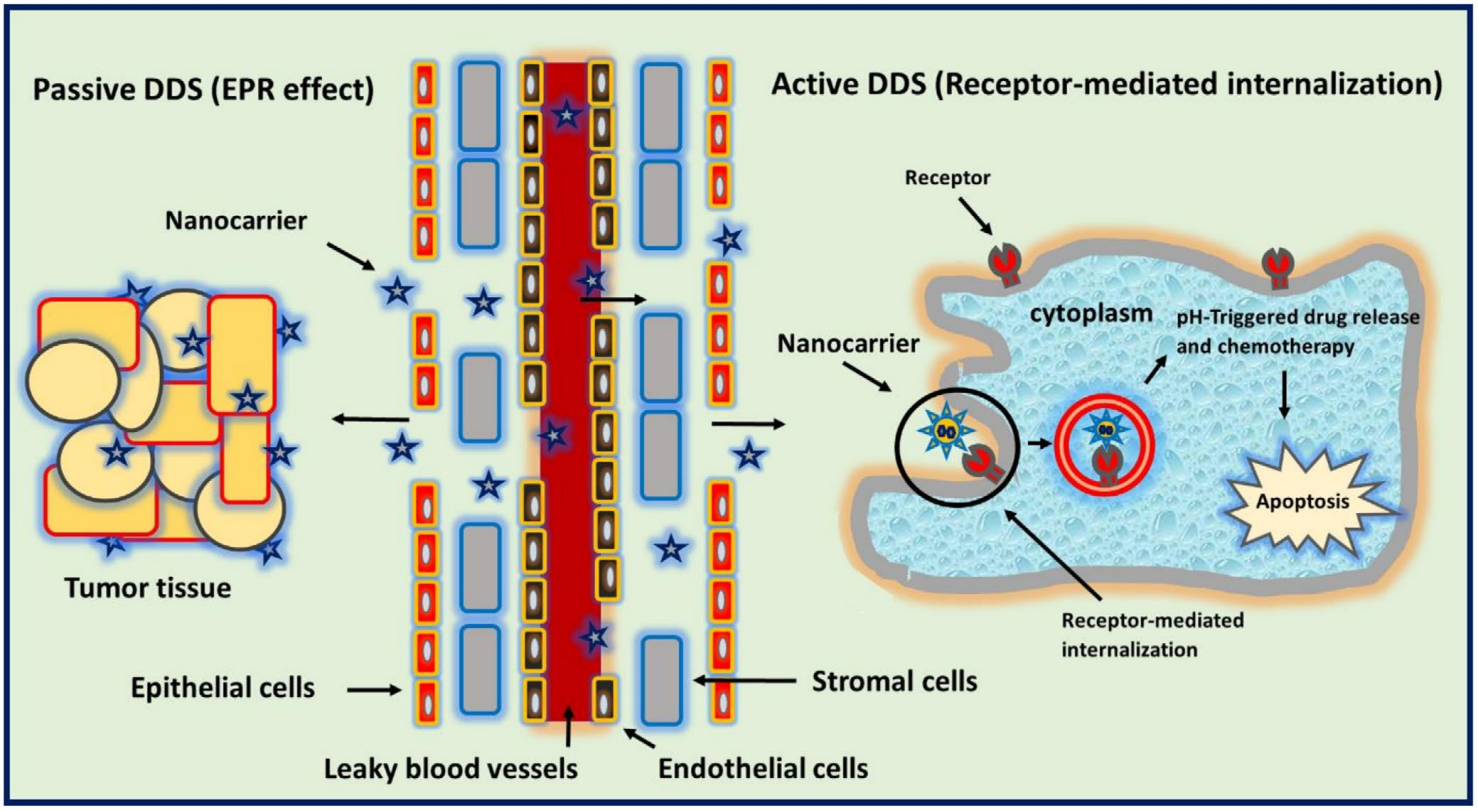
Passive and active DDSs based on polysaccharides.

**FIGURE 3:**
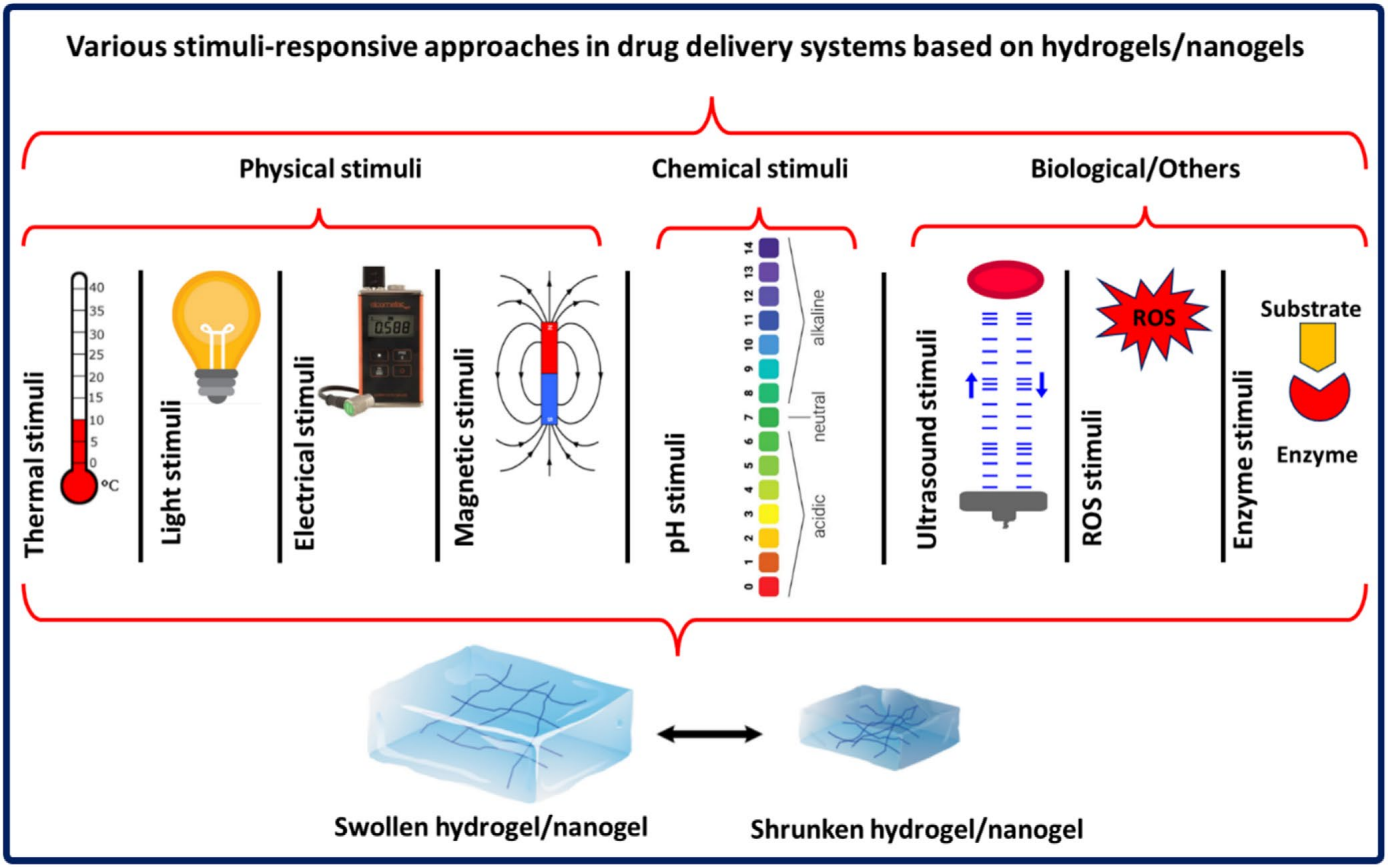
Stimuli‐responsive hydrogels/nanogels for polysaccharide‐based materials. These hydrogels/nanogels may be applied for drug delivery and tissue engineering goals.

There are two types of DDSs based on polysaccharides: passive DDSs (called non‐targeted DDSs) and active DDSs (known as targeted DDSs) (Figure 2) (Faramarzi et al. 2020; Rezvani et al. 2018; Narmani, Mohammadnejad, et al. 2019). In passive DDSs, the penetration of polysaccharide‐based DDSs can take place due to the presence of leaky vasculatures in disease tissues and result in the enhanced permeability and retention (EPR) effect. The EPR effects facilitate the penetration of DDSs in the site of action and enhance the drug bioavailability and drug release in the surface/near target cells (Ramadan et al. 2025; Saadh et al. 2024; Moutabian et al. 2022). These DDSs can also internalize into cancer cells through various types of endocytosis based on the size distribution. On the other hand, when the surface of polysaccharide‐based DDSs is functionalized with a targeting ligand, DDSs can be attached to their specific receptor on the surface of target cells, and receptor‐mediated internalization of DDSs is done. This strategy in disease treatment is called active DDS (Ramezani et al. 2024; Sivakumar et al. 2025; Narmani, Jahedi, et al. 2023; Narmani et al. 2020). The targeting ligands include bioactive/biomolecules, such as folic acid, peptides, antibodies, aptamers, etc., which can bind to their specific receptors on the membrane of target cells. There is another strategy in the delivery of drugs, which can be categorized as active DDSs (Narmani, Farhood, et al. 2018; Narmani and Jafari 2021; Narmani, Rezvani, et al. 2019). This strategy is nominated for stimuli‐responsive DDS in which polysaccharide‐based DDS responds to external/internal stimuli to reach the target site or to release the drug at the site of action. These stimuli‐responsive DDSs are divided into three types: biological stimuli‐responsive (glucose, enzyme, etc.), chemical stimuli‐responsive (pH, redox, etc.), and physical stimuli‐responsive (temperature, magnetic, etc.) DDSs (Narmani, Farhood, et al. 2018; Kolipaka et al. 2024; Wang, Nie, et al. 2025). Table 4 represents the practical examples of polysaccharide‐based DDSs. Figure 3 presents the scheme of stimuli‐responsive hydrogels/nanogels in polysaccharide‐based DDSs.

**TABLE 4:** The pharmaceutical (drug delivery systems) and biomedical (regenerative medicine) applications of polysaccharide‐based materials.

Polysaccharides	Therapeutics	Diseases/cancers	Comments	References
Drug delivery systems				
Chitosan‐bismuth oxide (Bi_2_O_3_)	Aminolevulinic acid, curcumin, Au nanoparticles	Breast cancer	Biodegradability, stability, high loading capacity, theranostics, and CT imaging	Dastgir et al. (2024)
Chitosan‐hairpin DNA (hpDNA)	Fluorescent hpDNA, Au nanoparticles	Breast cancer	Biocompatibility, theranostics, tumor‐related miRNA imaging, serum stability, and photothermal therapy (PTT)	Dong et al. (2023)
Alginate‐chitosan‐gelatin	Doxorubicin, Au nanoparticles	Breast cancer	Bioavailability, theranostics, pH‐sensitive drug release manner, and reliable mechanical durability	Ziaei et al. (2023)
Hyaluronic acid dialdehyde‐chitosan	siRNA	Bladder cancer	Serum stability, internalization capacity, controlled drug release, and CD44 targeting	Liang et al. (2021)
Cyclodextrin‐polyethyleneimine‐graphene quantum dot	miR21a	Liver cancer	Serum stability, high stability, theranostics, and high cellular uptake	Heidari et al. (2023)
Cyclodextrin‐polyethylenimine‐dextran‐5‐dithio‐(2‐nitrobenzoic acid)	Lonidamine	Various cancers	GSH‐sensitive DDS, ROS induction, MDR capability, and controlled drug release of bioactive	Tang et al. (2023)
Cyclodextrin‐cholesterol	5‐Fluorouracil, methotrexate	Breast cancer	Biodegradability, biocompatibility, serum stability, solubility, sustained drug release, and combinational therapy	Almawash et al. (2023)
Pectin‐β‐lactoglobulin	5‐Fluorouracil, palladium family drugs	Colorectal cancer	Efficient co‐delivery, combinational therapy, prolonged circulation, and high drug entrapment capacity	Leilabadi‐Asl et al. (2023)
Pectin‐mannose‐metal–organic framework	Methotrexate	Colorectal cancer	Targeted drug delivery, biocompatibility, high stability, and high drug entrapment and sustained drug release	Poursadegh et al. (2024)
Pectin‐oleic acid	Chlorogenic acid, iron oxide nanoparticles	Colorectal cancer	Dual‐targeted oral administration, co‐delivery system, potential cellular uptake, and bioavailability	Zhu et al. (2024)
Chondroitin sulfate	Antiangiogenic peptide	Various cancers	Antiangiogenic drug carrier, prolonged circulation time, high cancer cell targeting ability, ability to induce ROS and apoptosis, and in vitro and in vivo application	Li, Fu, et al. (2024)
Starch‐polyvinyl alcohol‐ graphitic carbon nitride	Doxorubicin	Breast cancer	Serum stability, pH‐sensitive carrier, apoptosis‐inducing activity, and sustained drug release	Alipournazari et al. (2024)
Starch‐polyvinyl alcohol	Quercetin, iron oxide nanoparticles	Liver cancer	Biocompatibility, biodegradability, serum stability, pH‐sensitive drug release manner, high drug loading capacity, and controlled drug release	Asl et al. (2024)
Starch‐alginate	5‐fluorouracil, curcumin	Colorectal cancer	Biocompatibility, pH‐responsive carrier, and high drug entrapment capacity	Pooresmaeil and Namazi (2023)
Regenerative medicine				
Chitosan	Cefotaxime sodium	Diabetic burn wound healing	Hydrogel membrane, ionically crosslinked with carrageenan and polyvinyl alcohol	Khaliq et al. (2022)
Chitosan	Teicoplanin	Antibacterial wound healing	Nanofibrous mats, blended with polyethylene oxide	Amiri et al. (2020)
Hyaluronic acid	Self‐healing tyramine‐modified hyaluronic acid	Soft tissue engineering	Hydrogel, visible‐light and enzymatically‐crosslinked, containing phenol/tyramine/gelatin‐ modified methacrylate 3D printing	Simińska‐Stanny et al. (2023)
Chondroitin sulfate	Combretastatin A‐4 phosphate	Disease therapy	Nanofibrous mats applied for drug delivery and regenerative medicine, blended with polyvinyl alcohol and crosslink with glutaraldehyde	Guo et al. (2016)
Ulvan	Non, mimicking the extracellular matrix	Bone tissue engineering	Sponge‐like scaffold, crosslinked with gelatin, platform for culturing human adipose‐derived mesenchymal stem cells	Tziveleka et al. (2020)
Alginate	Ciprofloxacin hydrochloride	Regenerative medicine	Nanofibrous mats blended with poly ethylene oxide and Triton X‐100 or Pluronic F‐127, by physical crosslink (CaCl_2_) procedure	Kyzioł et al. (2017)
Alginate	Non, mimicking the extracellular matrix	Vascular tissue engineering	Hydrogel, prepared by physical crosslinking based on CaCl_2_	Sousa et al. (2021)
Carrageenan	Non, mimicking the extracellular matrix	Bone tissue engineering and wound healing	Nanofibrous mats, blended with chitosan and polycaprolactone	Vargas‐Osorio et al. (2022)
Cellulose	Non, mimicking the extracellular matrix	Tendon tissue engineering	Nanofibrous mats, wet‐spinnability and crosslinked alginate/hydroxyethyl cellulose	Hojabri et al. (2023)

### 7.2 Regenerative Medicine

#### 7.2.1 Polysaccharides in Wound Healing

Wound healing is one of the prominent applications of polysaccharides in regenerative medicine. Injuries mainly occur in various tissues (especially skin), which can be healed through five consecutive steps: homeostasis, inflammation, migration, proliferation, and maturation. Skin wounds are divided into two types: chronic (with a healing period of 5 months to above 1 year) and non‐chronic (with a healing period of 8 to 12 weeks) (Varaprasad et al. 2020). The length of the healing time is affected by environmental factors (correlated with chemical, physical, irradiation, or thermal effects), age, injury location, and patient health. Implementing supportive operations may prevent the progression of injuries and appearance of infection in the wound site (Tang et al. 2025). Therefore, there is a fundamental requirement for the development of practical tools with supportive operation in wound healing. Polysaccharide‐based materials are mentioned as potential candidates for wound healing since they have indicated high biocompatibility, bioavailability, anticoagulant, hemostatic activity, controlled bioactive/growth factor release, and antibacterial function (Teymoorian et al. 2024). These biopolymers can preserve the hydration of injured tissue and prevent microbial infections at the site of action. The hydrogel platforms of polysaccharides are applied for wet wound healing, which could provide enough moisture in the damaged tissues, absorb exudates of the wound, and preserve tissue hydration for fast and efficient healing. Compared with other types of wound healing, mechanical strength, biodegradability, permeable texture to gas exchange, affordability, ease of use, and flexibility are mentioned as appropriate properties of polysaccharide‐based materials in wound healing. Lack of strong interactions between the surface of the damaged tissue and polysaccharide‐based materials not only may reduce the pain of the damaged site but also facilitate the self‐healing performance of tissue by supportive function (Zhao, Qian, et al. 2024; Mao et al. 2025). Table 4 presents the examples of polysaccharide‐based materials in wound healing applications.

#### 7.2.2 Polysaccharides Scaffolds in Tissue Engineering

**FIGURE 4:**
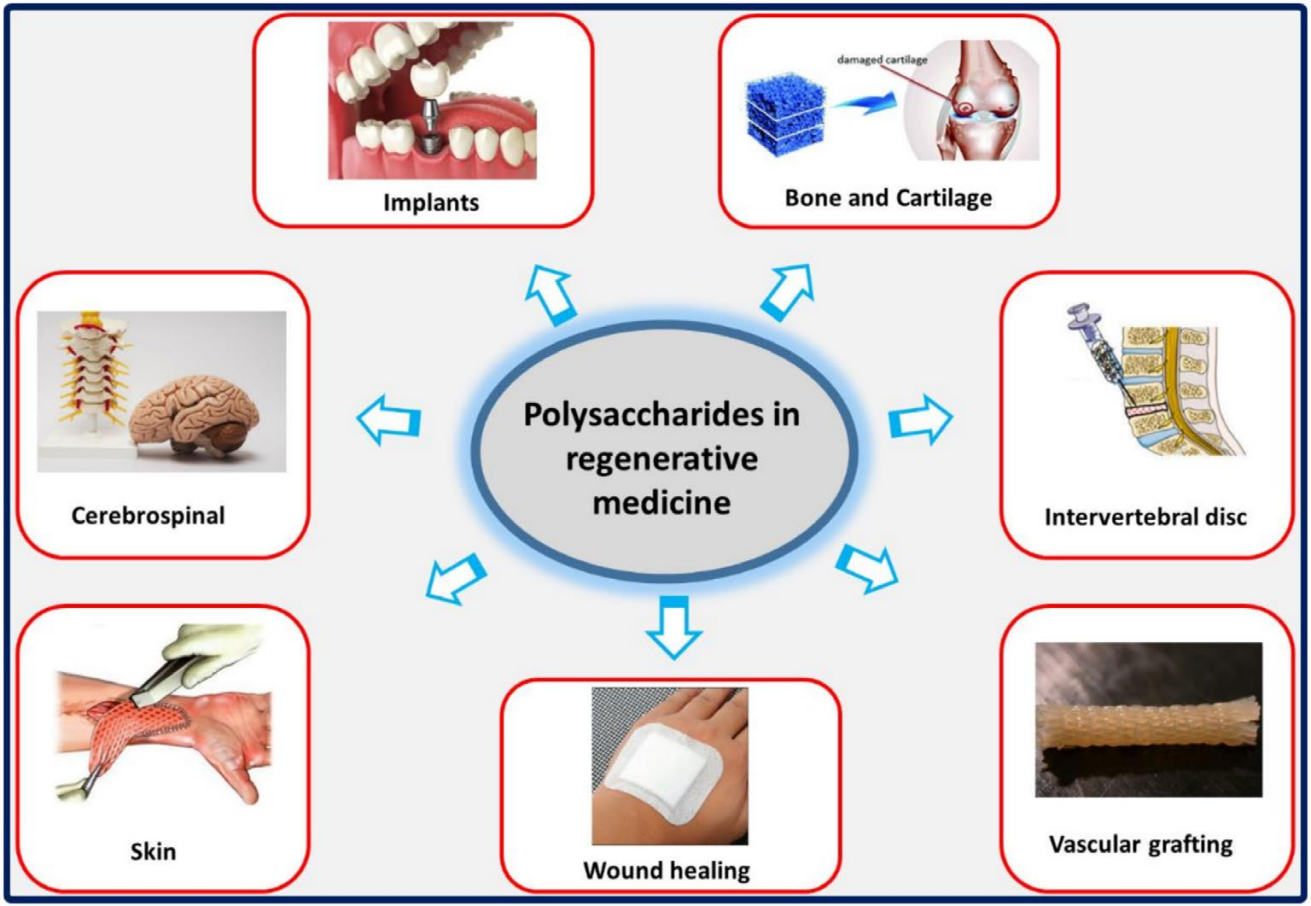
Applications of polysaccharides in regenerative medicine.

Polysaccharides have several considerable features that have made them reliable materials for tissue engineering (Figure 4). These aspects include surface engineering (modification and functionalization), mechanical durability, hydrophilicity, non‐immunogenicity, porosity, antimicrobial activity, biocompatibility, etc. (Alizadeh et al. 2024). Moreover, these biomaterials are applied for the development of physically assembled 3D scaffolds and hydrogels to be used in tissue engineering and repairing various tissues, like skin, bone, cartilage, implants, and cardiovascular tissues (Chinta et al. 2025). These biopolymers may efficiently provide reliable platforms for cell culture and mimicking extracellular matrix composition (containing fibrillar proteins like collagen, fibronectin, elastin, laminin, glycosaminoglycans, and proteoglycans), which would be beneficial for artificial tissue and organ development in in vitro and in vivo conditions. These scaffolds/hydrogels are embedded with metabolites, growth factors, and various bioactive materials to provide appropriate platforms for cell culture, growth, division, differentiation, orientation, and migration (Bhattacharyya et al. 2024).

The presence of functional groups on the surface of polysaccharides, like amino, carboxyl, and hydroxyl groups, facilitates the anchoring of target cells on the polysaccharide scaffolds since these positively and negatively charged functional groups may serve as negatively charged biomolecules (like collagen and glycosaminoglycans) and positively charged biomolecules (like elastin) of the extracellular matrix (Wang, Li, et al. 2025). Besides, the structural integrity, biodegradability, release rate of entrapped bioactive, and porosity of scaffolds/hydrogels are correlated with these electrostatic forces. To prepare highly durable scaffolds for bone and cartilage tissue engineering, polysaccharides combine with mechanically stable polymers (like poly(ε‐caprolactone), polyglutamic acid, polylactic‐co‐glycolic acid, polyethyleneimine, etc.) (Sivakumar et al. 2023). The conductivity, porosity, surface hydrophilicity, durability, roughness, thermal resistance, surface functional groups, swelling/deswelling manner, and hydrophilic‐hydrophobic balance are other characteristics that alter the function of polysaccharide‐based platforms. The polysaccharide‐based scaffolds are widely developed for the fabrication of artificial medical implants, which can be replaced with injured tissues/organs, like muscles, vasculatures, cartilage, bone, periodontal, and cerebrospinal tissues (Sivakumar et al. 2023; Sanjanwala et al. 2024). Table 4 demonstrates several practical examples of polysaccharides in tissue engineering.

### 7.3 Biosensors

Prognosis, diagnosis, and detection of diseases have an important role in the survival rate of patients. The early detection of disease can also save the investments and costs on health science; as a result, it has equal importance in disease treatment (Amini et al. 2019). Biosensors are sensitive analytical tools for the accurate and fast detection of disease biomarkers/chemical elements, such as heavy metals, glucose, cholesterol, and pathogens (Khalifa et al. 2023; Narmani, Kamali, Amini, Kooshki, et al. 2018). According to the definition, a biosensor is an analytical electronic tool that can detect biomarkers/chemical elements based on receptor‐target analyte interactions and produce an electronic signal detectable by a detector. Biosensors have been applied for reliable diagnosis and detection of diseases (like cancers, infectious diseases, cardiovascular disorders, diabetes, and so forth), environmental monitoring (toxic elements), and food safety at molecular and cellular levels (Xiong et al. 2025). The fabrication of sensitive biosensors requires specific physicochemical properties, inducing stability in hydrophilic and hydrophobic solutions, ease of manipulation, a wide range of molecular weights, available functional groups, conductivity, Tg temperature, and structural durability, which are found in polysaccharides (Azari‐Anpar et al. 2023; Naderlou et al. 2020). Moreover, hemocompatibility, biocompatibility, and the capability to bind target molecules are biological aspects that pave the way to the use of polysaccharides for the fabrication of sensors, especially for the receptor and transducer parts. Immobilizing on a transducer surface (via chemical, physical, and crosslinking forces), providing a sensitive platform for biomarkers/biomolecules, and detecting the signals of the transducer are useful applications of polysaccharides in biosensors (Wang, Ma, et al. 2023). Polysaccharides may serve as bioreceptors for some biomarkers as well; for example, hyaluronic acid is a specific bioreceptor of the CD44 marker in cancer cells. Sensors based on polysaccharides have indicated an appropriate limit of detection (LOD), limit of quantification (LOQ), high specificity, reliable sensitivity, and desirable selectivity (Kim et al. 2025). Table 4 shows the examples of sensors based on polysaccharides.

## 8 Future Perspectives

Polysaccharides are the most abundant biopolymers in nature. These biomaterials have indicated potential applications in the food industry (diet, nutrition, and food additives) and health sciences (pharmaceutical and biomedical fields). However, there are some challenging issues correlated with these biomaterials in the above‐mentioned fields that should be addressed in future works. In what follows, these concerns are going to be explored: (i) The physicochemical properties of polysaccharides, such as molecular weight, functional groups, structural changes, viscosity, surface functional groups, changing shapes, etc., are the potential issues that should be surveyed in future studies. Further studies must be carried out to investigate the chemical structure of active polysaccharides and their antifatigue impacts on the human body. The breakthrough technologies must be applied for the analysis and separation of polysaccharides in terms of their physicochemical and electromechanical features to find a more reliable relationship between polysaccharides and their biomedical functions. (ii) Finding molecular mechanisms between the function of polysaccharides and immunoregulation, suppression of cancer in vivo, and composition of microbiotas and their metabolites would be beneficial in the assessment of polysaccharide activities in the food and health sciences. The mechanisms of polysaccharides in the regulation of microbiota and the impact of types, structures, and compositions on microbiota are other prominent issues that should be taken into consideration. The correlation between the impact of the safety of polysaccharide‐based diets and intestinal microecosystems under healthy circumstances must be investigated. The effects of polysaccharides on changing intestinal microflora and altering signaling pathways must be determined. (iii) The molecular mechanism of action of polysaccharides in ameliorating fatigue should be further assessed since it can serve as a potential nutritional additive for individuals with extreme physical activities in their daily lives and even athletes. Polysaccharide‐based diet may guarantee the future of food and health sciences. (iv) The solubility of hydrocolloids is an undeniable part of biomaterial in food and health sciences, which should be improved in polysaccharide‐based materials. Their surface functional groups (such as hydroxyl, carboxyl, and amine) are not enough to increase their hydrophilicity. This deficiency could be solved by their surface modification and functionalization with hydrophilic molecules/functional groups. (v) The mechanical characteristics (such as tensile strength, elasticity, etc.) of polysaccharides must be boosted by their surface modification with mechanically stable polymers, such as polycaprolactone, as it is a crucial factor for the fabrication of scaffolds/hydrogels and even micro/nano‐size drug carriers. (vi) The cost of extraction, separation, and fabrication of scaffolds/hydrogels/carriers based on polysaccharides is another issue that restricts the applicability of these biopolymers in clinical studies.

## 9 Conclusion

Polysaccharides are the most abundant biopolymers in nature. These renewable biomaterials have a variety of potential physicochemical characteristics that make them potential candidates for numerous nutritional, pharmaceutical, and biomedical applications. In comparison to chemo‐polymers, which are a kind of synthetic material, polysaccharides have a number of advantages, like biocompatibility, biodegradability, bioavailability, affordability, hydrophilicity, serum stability, etc. They are classified into several types based on their origin: plant, seaweed, animal, and microbial polysaccharides. They can be extracted via a number of advanced techniques that pave the way for their accessibility and applications for a wide range of applications, from the industrial sector to biomedical sciences. Anti‐inflammatory, immunomodulatory, hypoglycemic, hypocholesterolemic, anticoagulant, antiviral, antimicrobial, and antioxidant functions are mentioned as the most significant biological properties of these biomaterials. These features of polysaccharides have made them considerable biomaterials in food science, such as antifatigue function, nutritional elements for gastrointestinal microbiota, and providing health for the gut in living organisms. Besides, these biomaterials have been applied for pharmaceutical (drug delivery systems) and biomedical (regenerative medicine) applications.

## Author Contributions

**M V N L Chaitanya:** conceptualization (equal), investigation (equal), writing – original draft (equal). **Bahjat Alhasso:** data curation (equal), investigation (equal), writing – original draft (equal). **Wadhah Hasan Alkhazali:** data curation (equal), investigation (equal), writing – original draft (equal). **Ashok Kumar Bishoyi:** data curation (equal), investigation (equal), writing – original draft (equal). **Rami Oweis:** data curation (equal), investigation (equal), writing – original draft (equal). **S. Renuka Jyothi:** data curation (equal), investigation (equal), writing – original draft (equal). **Rishiv Kalia:** data curation (equal), investigation (equal), writing – original draft (equal). **Laxmidhar Maharana:** data curation (equal), investigation (equal), writing – original draft (equal). **Ashish Singh Chauhan:** data curation (equal), investigation (equal), writing – original draft (equal). **Hayder Naji Sameer:** data curation (equal), investigation (equal), writing – original draft (equal). **Ahmed Yaseen:** data curation (equal), investigation (equal), writing – original draft (equal). **Zainab H. Athab:** data curation (equal), investigation (equal), writing – original draft (equal). **Mohaned Adil:** data curation (equal), investigation (equal), writing – original draft (equal). **Asghar Narmani:** conceptualization (equal), project administration (equal), supervision (equal), validation (equal), writing – review and editing (equal). **Bagher Farhood:** conceptualization (equal), project administration (equal), supervision (equal), validation (equal), writing – review and editing (equal).

## Funding

The authors have nothing to report.

## Conflicts of Interest

The authors declare no conflicts of interest.

## Data Availability

The authors have nothing to report.
